# Exsection of the Head of the Femur and Removal of the Upper Rim of the Acetabulum, for Morbus Coxarius, with Perfect Recovery

**Published:** 1855-02

**Authors:** 


### ECLECTIC DEPARTMENT,

### AND SPIRIT OF THE MEDICAL PERIODICAL PRESS.

*Exsection of the Head of the Femur and Removal of the Upper Rim of the Acetabulum, for Morbus Coxarius, with perfect recovery.* By Lewis A. Sayre, M. D., Surgeon to Bellevue Hospital, etc.

On the 30th of March, 1854, I was called, in consultation with Dr. Throckmorton, to see Ellen G., 297 5th street, aged 9 years, who had been suffering for 18 months with morbus coxarius of the left hip, which was supposed to have resulted from a fall. She had been treated with issues, blisters, etc., together with the general tonic and anti-scorbutic remedies adapted to such cases; but the disease continued to progress, until an abscess was discovered, involving the whole upper front and inner portion of the thigh, accompanied with repeated chills, profuse sweats, and great prostration.

When I first saw her, this abscess had pointed in two places, and was apparently just ready to open; the point nearest the surface and most fluctuating was just by the anterior superior spinous process of the ileum, immediately in contact with the attachment of the tensor vaginæ femoris muscle, and Poupart's ligament. The other place of pointing was about five inches below the ligament, just over the femoral artery; pressure on any part of the upper portion of the limb distended both of these pointing abscesses, showing communication between them.

The leg was shortened 2¼ inches, and turned inward, *but not permanently fixed in its position*, (as is usual,) but allowing of considerable motion, which gave a distinct *bony crepitus* between the femur and ileum. The pelvis was twisted and drawn upwards. Her general health had become much affected, having lost her appetite, and she was suffering from hectic, with constant chills and profuse sweats, and was only rendered comfortable by the constant use of anodynes.

I advised a free opening of the abscess, and, if necessary, to remove the head of the femur. At first this was objected to; but, as the child's health rapidly failed and death seemed inevitable, the father, in a few days, consented to the operation. Accordingly, on the 29th of March, 1854, assisted by Drs. Throckmorton, Drake, Thebaud, Baeur, and Bertholf, I proceeded to perform it.

I first laid open the abscess by a free incision of about six inches, over the trachanter major, on the outer aspect of the thigh, and in a line with the femur, and then cut into the floor of the abscess (which principally occupied the inner and front portion of the thigh,) and discharged about a pint of thin serous and flaky pus. The finger was then readily passed around the neck of the femur, and detected an opening in the capsular ligament on the inner surface of the neck. The upper border of the acetabulum had been absorbed, and the head of the femur was upon the dorsum of the ileum, near the anterior superior spinous process, *surrounded by its capsule*, (which seemed to have been slipped up,) and a large deposit of bone, apparently being an attempt of Nature to make a new acetabulum. But this cavity thus formed had no lining membrane, as the femur grated roughly upon it. I then opened the capsular ligament on a line with the external incision, and disarticulated by bringing the leg strongly across the opposite thigh, and then with a large pair of Luer's forceps, readily cut off the head of the femur at the lower extremity of the neck. The bone at this point appeared perfectly healthy. I was very cautious not to injure the insertion of the psoas-magnus, or iliacus-internus, or any of the rotator muscles, which are inserted just behind the trochanter major.

The upper rim of the acetabulum had been absorbed, (according to the theory of Dr. March, of Albany,) and the new deposit of bone, which was intended to supply its place, was denuded and carious. I gouged it off with a sharp, firm chisel, made for that purpose, and, in this way, took off a number of flakes of bone, until I came to a healthy, bleeding surface.

The anterior superior spinous process on its outer surface, and the external lip of the crest of the ileum, was black and carious for some distance, and with the forceps I easily clipped it off until I came to a healthy bone. Very little blood was lost in the operation, and after cleaning away all the debris, I brought the leg in the straight position, filled the wound with lint, and dressed with a roller and cold water compress. She was then put to bed, and a cup of strong coffee administered, after which she soon fell asleep.

The child was under the influence of chloroform during the operation, which occupied nearly 20 minutes, and was perfectly insensible the whole time.

The following extracts from my note book, taken at each daily visit, exhibit the progress of the case:

11 P. M.—Has slept occasionally and is quite comfortable; pulse 128; skin good; vomited freely about 4 P. M.

*March* 30, 10 A. M.—Passed a good night, without any narcotic, and slept about four hours; has had no chill; taken breakfast with a relish, and is surprisingly comfortable, considering the magnitude of the operation; pulse 120; no hemorrhage; passed urine twice.

*March* 31.—Took half a grain of opium last night; slept well; pulse 120; skin good; removed external layer of lint; found small amount of pus.

*April* 1.—Slight fever; heat of skin and thirst; pulse 130. Administered 5 gr. Dover's powder, with addition of half a grain ipecac., every four hours.

*April* 2.—Has passed a good night, slept six hours, ate a good breakfast, and feels every way better, but is much more feeble; dressed the wound; on removing the lint, found healthy pus in abundance.

The abscess, which pointed at the anterior superior spinous process, being again full and fluctuating, I opened it, and gave exit to about a tablespoonful of tolerably healthy pus; pulse 140, and more feeble; directed to administer brandy and beef-tea more liberally; I do not think the family give sufficient stimulant or nourishment, as they are very strongly opposed to brandy, and are afraid of meat on account of fever.

*April* 3.—Slept well all night without opiate; pulse 120; bowels moved twice naturally; appetite good; finding great improvement, follow a more nutritious diet; I advised its continuance.

*April* 4.—Same as yesterday; healthy suppuration, rather abundant.

*April* 5.—Child very comfortable, amusing herself by cutting paper dolls; applied the straight splint for counter extension to the well side, and made extension by means of the foot-board, bringing the limb down to the same length of the opposite one.

*April* 6.—Slept well; bowels moved naturally; but pulse more quick and feeble, 160; has not eaten so well; ordered brandy and soup to be given more liberally.

*April* 7.—Slept well, but much weaker, having had three loose discharges in the night, and some hemorrhage from the nose, which was arrested by astringents and compress. Ordered brandy and laudanum, with more liberal use of iron.

*April* 8.—Diarrhœa not yet checked; the brandy and opium was not given, and yet the child is somewhat stronger than yesterday; pus more consistent.

*April* 9.—Diarrhœa checked; slept well; eats freely; discharge less copious and more consistent; pulse 120.

*April* 10.—Very comfortable; looks as if it will require a counter-opening on the front of the thigh, at the old place of pointing.

*April* 13.—Doing well, and the wound filling with healthy granulations.

*April* 14.—I applied a compress and adhesive straps on the inside of the thigh.

*July* 1.—Dr. Throckmorton has seen the child daily since my last visit, and reapplied the bandage and compress, which has had a most salutary effect, and the abscess has the appearance of healing rapidly.

*July* 10.—I was again called to meet Dr. T. to-day, and found the child much prostrated from a severe attack of dysentery, which had lasted four or five days; she is very much reduced, and, I fear, will not rally. The granulations are flabby, and pus thin and copious.

*August* 1.—The dysentery has been checked for some days; but the wound, which was nearly closed, has opened, and a small piece of ragged bone came away, which was probably some portion of the shavings or chips removed from the ileum, at the time of the operation, and which I had not been sufficiently careful to remove.*

*August* 20.—The child very much improved, but the fistulous opening, from which the piece of bone had escaped, remaining, and having rather a white and flabby appearance, I injected it with tinct. iodine.

*August* 24.—The injection has been followed by a smart attack of erysipelas, which has extended down some distance below the knee, and there is considerable constitutional disturbance.

*Sept.* 1.—The erysipelas gradually subsided, but seems to have been of great service, as it has caused union of the walls of the abscess all around the thigh, and the small opening in the cicatrix is nearly closed, discharging a very few drops of healthy pus. The limb is still in the extending splint; but on removing it there seemed no tendency to retraction of the limb. The splint was reapplied; but the body was left free from the bandage, so as to allow of flexion in order to prevent enchylosis.

I might here mention, that for some weeks past, since about the 1st of August, at each dressing her body has been brought at a right angle with the thighs, having this object in view; and I have now permitted her to do it as often as she likes.

*Nov.* 1.—I had not seen the case for two months, until to-day, when to my astonishment, I found her walking on her crutches, which she has been able to do for some two weeks. Her limb appears the same length as the other, and she can flex and rotate it freely. I directed her to bear no weight upon it yet.

*Nov.* 20.—To-day I placed her in the horizontal position, and measured her carefully, and find there is about $\frac{1}{8}$ or nearly $\frac{1}{4}$ of an inch shortening. By taking hold of the foot, the whole body can be drawn down in the bed without pain in the joint, and a pressure may be made sufficiently strong to move the pelvis and body upward without producing any shortening of the limb. When she lies upon the back, with the leg extended upon the thigh, she can elevate the heel sixteen inches from the bed, and flex the knee so as to bring the thigh at a right angle with the pelvis; she can rotate it internally so as to touch the other foot, and externally so as to touch the bed. Her general health is perfect, and the case has terminated perfectly successfully. I feel in duty bound to express, in this place, my warmest thanks to Dr. Throckmorton, for his constant care and attention of this interesting case, and to which I am confident I am greatly indebted for so successful a result,

---

* Since making this note, my impressions have been more confirmed, as two similar pieces of bone have been removed from different parts of the cicatrix, and have thus materially retarded the progress of the case; I should therefore advise great care, after the performance of this operation, that all debris and foreign bodies be carefully washed from the wound; and in so large and ragged an abscess as this one was, it will require more care than any one would imagine, unless they had seen it.

as the distance from my house rendered it impossible for me to give it the care required.

The bone was carefully examined, microscopically, but no trace of tubercle was found.

Remarks.—The history of exsection of the head of the femur in hip-joint disease lies within the present century. The first surgeon who suggested the possibility of exsection of this bone, was Mr. Charles White, in 1769; but the first to attempt its performance, in morbus coxarius, seems to have been Schmalz, in 1816. In this case the head of the bone was found loose, and simply required removal; but it was, on this account, no less a case of exsection in this disease, and its successful results add much interest to the early history of the operation. In 1818, Anthony White performed his celebrated operation, which has generally been referred to as the first successful attempt to exsect the head of the femur in morbus coxarius. It seems to have been repeated in Great Britain but once, and then by Hewson, of Dublin, until 1845, when Mr. Fergusson operated successfully. Since this date it has been very frequently performed in England; and within the last year we find notices of three operations in the London Hospital. Mr. Fergusson has operated five times, and, as far as we can learn, with uniform success; one patient died two years after the operation, of "enlargement of the liver, after having experienced great relief from the proceeding."

Mr. Fergusson states (*Med. Chir. Trans.*, vol. 28), that he has learned that Mr. Brodie performed this operation, and "the patient died within a few days after, the direct effect of that proceeding;" but Mr. Henry Smith, writing in 1848 (*London Lancet*), remarks that he has not been able to "obtain any accurate information respecting the correctness of this assertion." There is no doubt that this surgeon did exsect the femur at St. George's Hospital, about the year 1836, but under what circumstance it does not appear.

In this country this operation seems to have attracted little attention. A case is reported in the *N. Y. Med. Surg. Reporter*, Jan. 10, 1846, in which Dr. J. P. Batchelder, of this city, removed the head of the femur in 1845, under the following circumstances: A young man was kicked by a horse upon his hip, four or five years before, which gave rise to severe symptoms: fistulous openings formed and discharged pus freely; the probe finally detected bone; the fistula was dilated with sponge tents, and the dead bone removed with forceps, which proved to be the head of the femur; the patient now rapidly improved and eventually recovered.

I have learned that Dr. Parkman, of Boston, exsected this bone in 1853, but the particulars and results of the case I have no knowledge of. Dr. Bigelow, of the same city, operated soon after, but the case terminated fatally. From a somewhat extended examination of our medical periodicals, I have been unable to find records of any other case.

By a careful examination of the accompanying table I think the propriety of the operation will be admitted. We might maintain its importance, also, from the fact that it is but following the indications of Nature. The cases of Schmalz and Kluge, in which the head of the femur was found separated from the shaft, are examples of natural efforts to remove the foreign body. Two striking instances of this kind were reported to the *Dublin Pathological Society*, in 1839, by Dr. Carlile, who exhibited two specimens of the epiphyses of the femur, which had been spontaneously discharged in two cases of hip-disease. One patient was 20 years of age, the other 5; both rapidly and completely recovered.

In preparing the following table, regard was had to exsection only in morbus coxarius, as it is in reference to this particular class of cases that we wished to estimate the value of the operation. To arrive at the most satisfactory conclusion in regard to the propriety of the operation, each case ought to be carefully examined, and the complications duly considered. It will be sufficient, however, for our purpose, to notice briefly the fatal cases, and, as far as given, the causes of death; by these means we can estimate the part which the operative proceeding bore in the fatal issue of the several cases.

*Hewson's Case.*—Profuse suppuration; died three months after the operation; extensive disease of the cotyloid cavity found, with perforation of the acetabulum and formation of abscesses in the pelvic cavity.

*Textor's first Case.*—Sloughs formed on the sacrum; died on the 53d day after the operation; on examination, the wound was found nearly cicatrized; there was the commencement of a false joint, consisting in bony deposits on the femur, and a depression on the ileum.

*Textor's second Case.*—Gangrene of the wound took place, and the patient died on the 4th day.

*Fergusson's Case.*—Died two years after, of enlargement of the liver; wound never entirely healed, owing to a piece of necrosed bone from the edge of the acetabulum, which the trochanter major prevented from escaping. The operator remarks: "I left the trochanters, being under the impression that by taking away the diseased head of the femur only, I lessened the danger, but it was found in the after treatment, that the trochanter major so projected outward in the line of incision as greatly to retard the closing of the wound, and I had no doubt, on inspection after death, that it had acted as a kind of cap to the acetabulum, and prevented the necrosed portion of bone, above referred to, from getting out." *

*Roux's Case.*—Secondary hemorrhage; died on 7th day; post-mortem examination revealed a large collection of pus between the glutei muscles; extensive disease of the cotyloid cavity; extensive disease of femur below its section; pus in the medullary cavity, disease of pubic bones.

*Simon's Case.*—Particulars of this case not given.

*Smith's Case.*—Progressed favorably for a time; symptoms of Bright's disease made their appearance, and he died four and a half months after the operation. Kidneys found in an advanced stage of Bright's disease; sinuses were found extending upwards along the psoas muscles and originating in caries of the upper lumbar vertebræ.

*Hawkin's Case.*—Patient very much exhausted; suffered little by the operation; died on 3d day. On examination the acetabulum was found perforated so as readily to admit the passage of the finger.

*Bigelow's Case.*—Particulars not given.

*Erichsen's Case.*—Particulars not given.

It will be seen that, as far as the particulars of these fatal cases are given, death was attributable in most instances to some grave complication. The

* We do not learn that he used any extension—if so, this ought not to have happened.

operation has almost invariably produced the most favorable change in the general symptoms of the patient, and, in several cases, the parts involved in the operation have healed in the most satisfactory manner, when at length the patient has succumbed to some new and acute disease.

By a careful examination of the tabulated cases, the propriety of the operation must be admitted, and also the great importance of not neglecting it too long, lest the constitution becomes so exhausted as not to rally from the shock, or sinks from excessive suppuration. By too long delay, also, the acetabulum may become so much involved as to render the operation useless.

So soon as *suppuration* is formed *in the joint*, and the *synovial membrane* has been *destroyed*, which can be ascertained by *fluctuation* and *crepitus*, I should advise a *free incision* to be made *into* the joint, just posterior to the trochanter major, to give free exit to the pus. Extension should then be made, after the plan proposed by Prof. March, of Albany—otherwise the bones of the pelvis may become seriously involved. From personal experience in opening suppurative joints, I am satisfied that there need be no fear from the admission of air, which has always been so much dreaded. This fear has, doubtless, arisen from the great constitutional disturbance which follows its introduction into *healthy* joints, but we must recollect that after suppuration and destruction of the synovial membrane, that it has in fact no longer the *characteristics* of a *joint*, but is the same as any other abscess connected with bone, and should be treated upon the same general principles. By this prompt treatment in the *earlier* stages of the *suppuration*, we will avoid the necessity of *exsection*, and in the majority of cases have a speedy recovery with tolerable motion, and in many instances perfect and complete. Two such cases have occurred in my own practice, and I have seen a number in the practice of my friends. But as I did not intend to speak of suppuration of the joints in this paper, but simply of exsection for caries or necrosis, I will postpone the further consideration of this subject, merely remarking, that if we will carefully study the fatal cases which I have reported, I think we cannot help but infer that, had the plan proposed been earlier pursued in those cases, we might have anticipated a more favorable result, at least, in some of them.

SUMMARY.— *Whole number*, inclusive of above case, 30.

*Recovered*, 20.—Of these, 13 were completely successful; 3 died of an intercurrent disease, at periods varying from three months to two years after the operation; 1 is reported as not having progressed favorably; the remainder were too meagerly reported, or too recently performed to decide correctly of results.

*Died*, 10.—Of these, 4 died within one week after operation; 1 on the 12th day; 2 in two months; 1 in four and a half months; 1 some months after; 1 unsuccessful.

